# Vaccination risks in children with rare diseases

**DOI:** 10.1186/1824-7288-41-S2-A5

**Published:** 2015-09-30

**Authors:** Ignazio Barberi, Venera Tiralongo, Lucia Marseglia

**Affiliations:** 1 Neonatal Intensive Care Unit, Department of Pediatrics, University of Messina, Messina, 98125, Italy

## 

Infectious diseases are a major health problem worldwide, causing many serious complications, including autoimmune disorders in unvaccinated populations. The vaccinations represent the most effective method of preventing infectious diseases and cost benefit analysis of vaccination programmes demonstrated their utility. On the basis of Orphanet database, in Italy rare diseases affect almost 2 million people, and 70% of them are children. Among neurodegenerative disorders, children with spinal muscolar atrophy should receive routine immunizations, including influenza and pneumococcal vaccines; for pediatric patients affected by Becker's Muscular Dystrophy influenza and pneumococcal vaccines are indicated in case of severe weakness of respiratory muscles [1]. Immunocompromised patients are a unique group with special issues regarding immunization: in case of B and T cell immune deficiency, live attenuated vaccines (Oral polio vaccine, Measles, mumps, rubella, Bacillus Calmette-Guerin, Varicella Zooster) should be avoided, as the major risk of vaccine induced diseses [1]. Immunocompromised individuals have suboptimal response to Hepatitis B vaccine, therefore they should receive a double dose. The administration of vive vaccines is contraindicated in patients with agammaglobulinemia including X-linked agammaglobulinemia, hyper IgM Syndrome, and common variable immunodeficiency and in patients with Wiskott-Aldrich syndrome. Subjects with mitochondrial diseases should be vaccinated following the routine immunization schedule [1]. Vaccination with vive attenuate vaccines should be avoided in patients with rheumatologic disorders only if they are receiving high dose of immunosuppressive drugs [2]. Measles-mumps-rubella immunization is contraindicated in subjects whit autoimmune purpura as the greater risk of thrombocytopenia [3]. A special mention is reserved to premature infants. It is wrongly believed that in premies allogenic stimuli can be dangerous as these infants are weak and already stressed from resuscitation strategies and aggressive therapies. Moreover they present IgG deficit, as immunoglobulin cross placenta barrier mainly during the thirst trimester of pregnancy. However, it has been demonstrated that premature infants have a proper immune response to vaccinations received in the first year of life and side effects do not exceed that recorded for terms infants. Therefore, preterm babies should be vaccinated according to the recommended schedule for term infants, without correction for gestational age. In conclusion, immunocompromised subjects and premature infants do not have a higher incidence of adverse reactions following immunization if all preventive measures and recommendations are adopted [4].
